# Management of Intractable Nasal Hyperreactivity by Selective Resection of Posterior Nasal Nerve Branches

**DOI:** 10.1155/2017/1907862

**Published:** 2017-12-12

**Authors:** Daisuke Takahara, Sachio Takeno, Takao Hamamoto, Takashi Ishino, Katsuhiro Hirakawa

**Affiliations:** Department of Otorhinolaryngology, Head and Neck Surgery, Division of Clinical Medical Science, Programs for Applied Biomedicine, Graduate School of Biomedical Sciences, Hiroshima University, Hiroshima, Japan

## Abstract

The posterior nasal nerves emerge from the sphenopalatine foramen and contain sensory and autonomic nerve components. Posterior nasal neurectomy is an effective method to remove pathological neural networks surrounding the inferior turbinate that cause unregulated nasal hypersensitivity with excess secretion in patients with severe allergic rhinitis (AR). We describe the sophisticated endoscopic surgical procedure that allows feasible access to the confined area and selective resection of the nerve branches with the preservation of the sphenopalatine artery (SPA). We retrospectively analyzed the cases of 23 symptomatic severe AR patients who failed to respond to standard medical treatment and underwent surgery. There have been no major complications after surgery including nasal bleeding or transient numbness of the upper teeth. The mean total nasal symptom scores (TNSS) were decreased by 70.2% at 12 months after the procedure. Our comparison of the clinical effectiveness based on the number of severed nerve branches revealed that the improvement of the TNSS was significantly higher in patients with >2 branches. We conclude that this minimally invasive technique that preserves the SPA is clinically useful and decreases the rate of postoperative complications. This trial is registered with UMIN000029025.

## 1. Introduction

In patients with severe allergic rhinitis (AR) who are resistant to standard combinations of medical treatments, surgical treatment can be chosen as a successful alternative strategy. Over the last two decades, many endoscopic surgical techniques have been documented to alleviate nasal hyperreactivity accompanying severe nasal obstruction. Among them, posterior nasal neurectomy is an effective method to transect the neural networks surrounding the inferior turbinate that cause unregulated nasal hypersensitivity with excess secretion. The basic procedure is to selectively cut nerve bundles at the level of the sphenopalatine foramen (SPF) with a transnasal approach [1–3]. The nerve bundles consist of parasympathetic and sympathetic components of the vidian nerve and somatosensory fibers from the maxillary branch of the trigeminal nerve, and they are distributed in the nasal mucosa following the branches of the sphenopalatine vessels [4].

Since the posterior nasal nerve runs through the foramen accompanying the vessels, the question of how to best manage the sphenopalatine artery (SPA) has been a matter of debate [5–7]. Resection of the entire neurovascular bundle by using an ultrasonic coagulator or a coblation system is often performed, but concerns remain regarding possible postoperative bleeding and unfavorable changes in the physiological blood supply in the posterior part of the nasal cavity. Herein we describe the surgical procedure of our endoscopic technique using originally designed microinstruments that allows feasible access to that confined area and the selective resection of the nerve branches with the preservation of the SPA. We also assessed the clinical effectiveness of this procedure based on subjective nasal symptom scores in a series of retrospectively reviewed patients.

## 2. Patients and Methods

We analyzed the cases of 23 patients (16 males and 7 females) with perennial allergic rhinitis who had a history of allergic symptoms from 2014 to 2015. All of them had failed to respond to combinations of medical therapy or laser surgery and suffered excess nasal secretion and hypersensitivity. According to the Japanese guideline for AR [8], the degree of total severity of subjective symptoms in all our patients was either 3 (severe, *n* = 8) or 4 (very severe, *n* = 15). The inclusion criteria for allergic rhinitis in response to house dust mites* (Dermatophagoides farinae)* were determined by a positive skin test or a positive RAST score of class 2 or greater.

**Figure 1:**
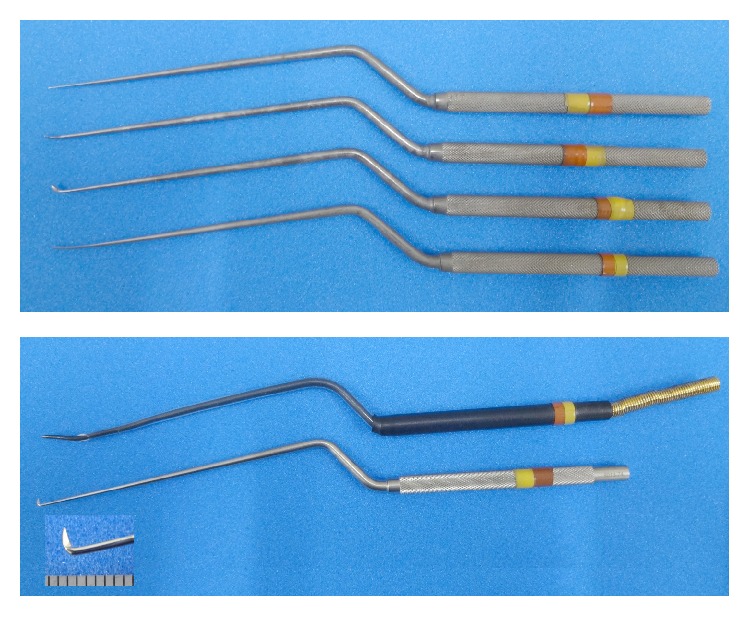
The microdissectors, malleable cauterization microprobe, and microknife.

After submucous inferior turbinectomy is completed by totally removing the turbinate bone, the uncinate process is removed and then the ostium of the maxillary sinus is widely opened to obtain wide exposure and visualization of the posterior lateral nasal wall with a 0-degree endoscope. A local injection of the lidocaine and epinephrine solution is made into this area to facilitate the dissection and hemostasis. Next, a vertical incision at the level of the posterior fontanelle is made and the mucosal flap is gently elevated posteriorly until the ethmoidal crest in the perpendicular plate of the palatine bone is reached. Gentle and wide elevation using a suction curette around the crest allows for better visualization of the neurovascular bundle emerging from the SPF. One may use the posterosuperior margin of the maxillary antrum as a reference point to locate the SPF. A set of microdissectors is used to dissect the bundle sheath and isolate the nerve branches from the adjacent vessels (Figure 1).

**Figure 2:**
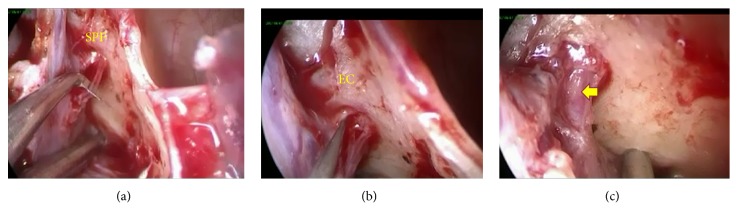
Intraoperative endoscopic view showing the branches of the posterior nasal nerve emerging from the SPF. (a) Resection of the anteroinferior branch toward the inferior turbinate. (b) Resection of the posterosuperior branch toward the middle meatus. (c) The SPA is well preserved after nerve resection (arrow). EC = ethmoidal crest.

A microknife (Fujita Medical Instruments, Tokyo), which is custom-shaped in its blade size down to two-thirds of the original length, is applied to have each nerve branch severed. Caution is taken to preserve the SPA in order to avoid massive bleeding. The posterior nasal nerve arising from the SPF is generally composed of two to four nerve branches on endoscopic examination. There are two major branches visible in most cases, that is, the anteroinferior branch toward the inferior turbinate and the posterosuperior branch toward the middle meatus. We make an attempt to sever all of the nerve branches that can be endoscopically identified (Figure 2). The number of nerve braches for each nasal cavity of the patient was recorded in order to evaluate the possible relation with clinical efficacy. After nerve sectioning, a malleable cauterization microprobe is used for capillary hemostasis and to prevent reinnervation. Finally, the mucosal flap is replaced to cover the remaining SPA, and nasal packing with absorbable gelatin sponge and PVA sponge (Merocel®, Medtronic Japan, Tokyo) is left in place for 1-2 days.

The study was approved by the ethical committee at Hiroshima University (number E-1958) and registered at the UMIN Clinical Trials Registry System (http://www.umin.ac.jp/ctr/index-j.htm, ID: 000029025). Written informed consent was obtained from all patients prior to surgery.

## 3. Results

The above-described procedure combined with submucous inferior turbinectomy has routinely provided clear visualization of the SPF area and allowed for dexterous dissection of nerve branches and the avoidance of vascular injury at the time of surgery. There was minimal coagulation near the incision in the lateral wall of the middle meatus after packing removal. Fibrin exudation and crusting nearly disappeared 1-2 weeks after surgery. There were no major complications after surgery including nasal bleeding that required nasal packing or transient numbness of the upper teeth.

**Figure 3:**
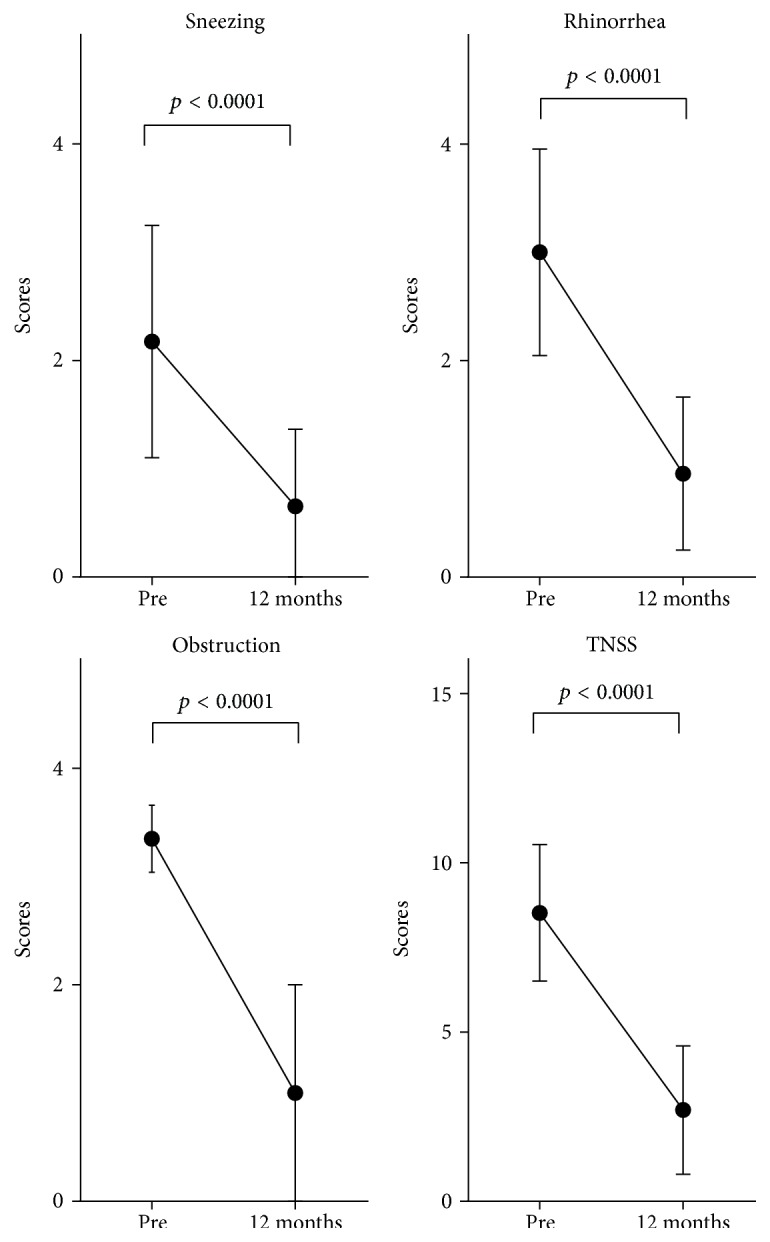
Changes in each nasal symptom and the total nasal symptom score (TNSS) after surgery (*n* = 23). The data were examined with the Wilcoxon signed rank test. Symbols and error bars represent mean values with standard errors. Pre = pretreatment.

We conducted a retrospective review of the patients' clinical records. Regarding clinical effectiveness, most of the patients reported subjectively excellent or good results. Subjective nasal symptoms (sneezing, rhinorrhea, and obstruction) were recorded and scored according to the Japanese guideline for allergic rhinitis [8]. Each symptom was graded according to the following 5-point severity scale: 0, none; 1, mild; 2, moderate; 3, severe; 4, most severe. The total nasal symptom score (TNSS) was calculated by adding the scores of the individual nasal symptoms. As shown in Figure 3, the mean symptom scores for sneezing, rhinorrhea, and nasal obstruction were all significantly decreased at 12 months compared to the preoperative baseline (*p* < 0.0001). The mean TNSS after the procedure also significantly decreased from 8.52 to 2.54 at 12 months, that is, by 70.2%. In addition, 39.1% (9/23) of the patients had remained almost free from all symptoms without medication at 12 months.

**Figure 4:**
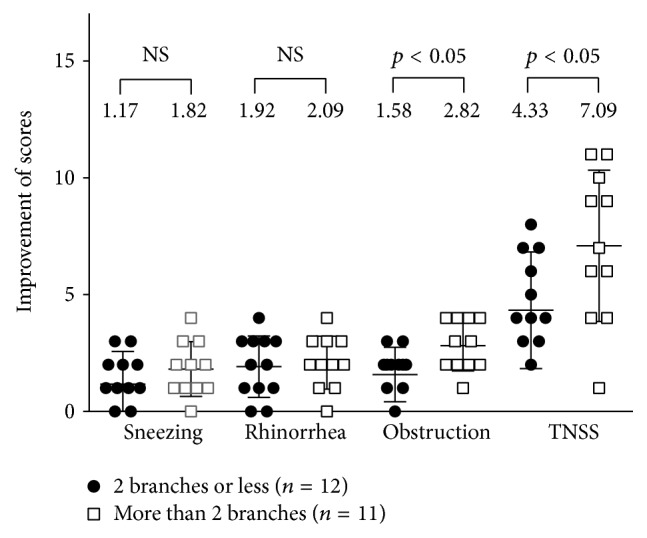
The improvement of each nasal symptom and the TNSS in the group of ≤2 branches versus the group of >2 after surgery. The data were examined with the Mann–Whitney* U* test. Bars and wings represent mean values with standard errors. NS = no significance.

Because the present technique provided us with the clear visualization and accurate dissection of nerve branches, we compared the clinical effectiveness between patient groups classified by the number of nerve branches severed for each nasal cavity of the patient: those with two branches or less (the ≤2 group) and those with more than 2 branches (the >2 group). As shown in Figure 4, the degrees of improvement of the scores for nasal obstruction and TNSS were significantly higher in the >2 group compared to those in the ≤2 group at 12 months.

## 4. Discussion

Surgical treatment for AR patients has been considered as a successful alternative strategy when the patients are resistant to standard combinations of medical treatment. According to the Japanese guidelines for AR, the objectives of surgical treatment for AR include modulation of the nasal mucosa, correction of the nasal cavity to improve nasal ventilation, and improvement of hyperreactivity with rhinorrhea [8]. Among the available surgical techniques, posterior nasal neurectomy is widely performed in Japan because of its clinical effectiveness for unregulated nasal hypersensitivity and because it is a minimally invasive endoscopic procedure.

The posterior nasal nerve emerges from the SPF and is distributed to the inferior turbinate mucosa following the branches of the sphenopalatine vessels. Innervation of the parasympathetic component increases the secretomotor function, and innervation of the sensory component regulates the sensitivity of the nasal mucosa [4, 9]. By resection of the posterior nasal nerve at this point, we can expect modifying the hyperreactivity of the neural network that augments the allergic reaction. In addition, this technique causes partial denervation of the middle turbinate and septum submucosal glands based on anatomical innervation [10]. Previous studies showed satisfactory clinical results of posterior nasal neurectomy for the most part, including more than 50% patients being almost totally free of nasal symptoms [2, 3, 11]. Kamijo et al. also reported significant improvements in symptoms related to quality of life, such as sleep disorder and malaise/feebleness after surgery [12]. In the present study, we also found that about 40% of the patients were relieved of subjective nasal symptoms at 12 months.

Several studies have provided histological and molecular evidence that posterior nasal neurectomy can lead to an attenuation of the orchestration of allergic inflammatory responses [2, 3]. Histopathological evidence in our earlier study revealed that the density of eosinophils and secretary glands was markedly reduced, accompanied by a decrease in the local IL-5 and eotaxin levels 6 months after surgery [2]. Resection of the posterior nasal nerve involved suppression of the secretagogue motor and the inhibition of neurogenic inflammation induced by parasympathetic and sensory denervation demonstrated by biopsy of the inferior turbinate 3-4 months after surgery [3].

There is a controversy regarding how to best manage the SPA in the process of nerve sectioning because of the anatomical configuration involved. Resection of the whole neurovascular bundle by using an ultrasonic coagulator or a coblation system has been used [7], but concerns remain regarding postoperative bleeding and unexpected deterioration in the physiological blood supply in the posterior part of the nasal cavity. In the present study, we introduced the use of microdissectors and a microknife to dissect the bundle sheath and to selectively resect isolated nerve branches, with the adjacent SPA kept intact. This less invasive technique with the aid of clear endoscopic visualization also provides an advantage regarding the patient's comfort. As for nerve resection techniques, we have found it easier and more efficacious to use the knife (i.e., hooking) than microscissors designed for pituitary surgery (i.e., pressing).

Another advantage of the present technique is to enable the accurate identification and dissection of nerve branches. We successfully severed the two major branches toward the inferior turbinate and the middle meatus in most cases. The degree of TNSS improvement was significantly higher in the >2 group as evaluated by the average number of nerve branches severed at surgery. All of these branches from the SPF are considered to relay autonomic and sensory innervation to the nasal mucosa of theinferior and middle turbinates in the lateral nasal wall [9]. However, further studies using objective parameters are required to determine whether the resection of these minor branches may have additional synergetic effects on severe neurological dysregulation.

We conclude that the selective resection of bilateral posterior nasal nerves is an effective technique in the management of intractable nasal hyperreactivity in severely allergic patients after 12 months of postoperative follow-up. This minimally invasive technique that preserves the SPA decreases the rate of postoperative complications. A limitation of this study is its relatively short follow-up period. Further studies with longer follow-up periods are required to test the long-term usefulness of this technique with the use of objective measures for assessing the main outcome.
